# Vitamin D status contributes to the antimicrobial activity of macrophages against *Mycobacterium leprae*

**DOI:** 10.1371/journal.pntd.0006608

**Published:** 2018-07-02

**Authors:** Elliot W. Kim, Rosane M. B. Teles, Salem Haile, Philip T. Liu, Robert L. Modlin

**Affiliations:** 1 Molecular Biology Institute, University of California, Los Angeles, Los Angeles, California, United States of America; 2 Division of Dermatology, David Geffen School of Medicine at University of California, Los Angeles, Los Angeles, California, United States of America; 3 Department of Hematology and Oncology, David Geffen School of Medicine, University of California, Los Angeles, Los Angeles, California, United States of America; 4 UCLA and Orthopaedic Hospital Department of Orthopaedic Surgery and the Orthopaedic Hospital Research Center, Los Angeles, Los Angeles, California, United States of America; 5 Department of Microbiology, Immunology and Molecular Genetics, University of California, Los Angeles, Los Angeles, California, United States of America; University of Tennessee, UNITED STATES

## Abstract

**Background:**

The immune system depends on effector pathways to eliminate invading pathogens from the host *in vivo*. Macrophages (MΦ) of the innate immune system are armed with vitamin D-dependent antimicrobial responses to kill intracellular microbes. However, how the physiological levels of vitamin D during MΦ differentiation affect phenotype and function is unknown.

**Methodology/principal:**

The human innate immune system consists of divergent MΦ subsets that serve distinct functions *in vivo*. Both IL-15 and IL-10 induce MΦ differentiation, but IL-15 induces primary human monocytes to differentiate into antimicrobial MΦ (IL-15 MΦ) that robustly express the vitamin D pathway. However, how vitamin D status alters IL-15 MΦ phenotype and function is unknown. In this study, we found that adding 25-hydroxyvitamin D3 (25D3) during the IL-15 induced differentiation of monocytes into MΦ increased the expression of the antimicrobial peptide cathelicidin, including both CAMP mRNA and the encoded protein cathelicidin in a dose-dependent manner. The presence of physiological levels of 25D during differentiation of IL-15 MΦ led to a significant vitamin D-dependent antimicrobial response against intracellular *Mycobacterium leprae* but did not change the phenotype or phagocytic function of these MΦ. These data suggest that activation of the vitamin D pathway during IL-15 MΦ differentiation augments the antimicrobial response against *M*. *leprae* infection.

**Conclusions/significance:**

Our data demonstrates that the presence of vitamin D during MΦ differentiation bestows the capacity to mount an antimicrobial response against *M*. *leprae*.

## Author summary

A key function of MΦ is to recognize, phagocytose and mount an antimicrobial response against microbial pathogens to defend the host. In humans, monocytes are recruited to the site of infection and differentiate into MΦ upon the onset of microbial infection. The MΦ phenotype and function are determined by the cytokine profile of the microenvironment in which the monocyte enters. Additionally, vitamin D is known to trigger direct antimicrobial responses against invading pathogens in MΦ, but also disrupts the differentiation of immune subsets within the myeloid lineage. Therefore, we investigated whether vitamin D status during MΦ differentiation influenced either phenotype or function. Here, we found that the IL-15 MΦphenotype is sustained regardless of vitamin D status. In contrast, antimicrobial MΦ differentiated in the presence of vitamin D exhibited a robust expression of an antimicrobial peptide, relative to MΦ differentiated in the absence of vitamin D. The antimicrobial MΦ armed with cathelicidin prior to *M*. *leprae* challenge demonstrated a strong antimicrobial response against the invading pathogen. Our study reveals that the presence of sufficient levels of vitamin D prior to microbial infection contributes to effectively reduce the viability of the pathogen in MΦ.

## Introduction

The MΦ is a sentinel of the innate immune system that serves as the first line of defense to recognize and destroy invading microbes. In human MΦ, activation by a toll-like receptor 2/1 (TLR2/1) ligand or interferon-γ (IFN-γ) triggers a direct antimicrobial response that depends upon the level of available vitamin D [1–3]. The vitamin D-dependent antimicrobial pathway involves the induction of IL-15 and IL-32, the conversion of 25D3 to bioactive 1,25-dihydroxyvitamin D (1,25D3) and subsequent activation of the vitamin D receptor (VDR) to induce the expression of the antimicrobial peptides including cathelicidin, autophagy and phagolysosomal fusion [2, 4–8]. This antimicrobial pathway is not induced in MΦ if the levels of 25D are not sufficient.

Macrophages demonstrate phenotypic heterogeneity which confer distinct functions in the innate immune system [9]. IL-15 MΦdemonstrate a vitamin D-dependent antimicrobial profile which includes the expression of CAMP mRNA [4, 10]. In contrast, primary human monocytes treated with IL-10 differentiate into phagocytic macrophages (IL-10 MΦ), which readily take up bacteria but weakly express the vitamin D-dependent antimicrobial pathway [10]. These MΦ subtypes can be identified by a specific cell surface phenotype, both IL-15 MΦ and IL-10 MΦ express CD209 but only IL-10 MΦ express CD163. As such, IL-15 MΦ and IL-10 MΦ are differentially identified in the polar forms of leprosy caused by the intracellular bacterium *M*. *leprae*, correlating with the different outcomes of infection.

In addition to its role in MΦ antimicrobial function, vitamin D has long been recognized to affect the differentiation of diverse cell types, including cells of the myeloid lineage [11]. Activation of the VDR converts malignant myeloid leukemia cells into non-proliferating monocytes or MΦ [12–15]. Dendritic cells differentiated in the presence of 25D3 or 1,25D3 demonstrate aberrant differentiation and decreased antigen presentation *in vitro* [16, 17]. MΦ differentiated in vitamin D have also demonstrated a change in phenotype and phagocytic function *in vitro* [15, 18]. Most of these studies were performed by adding non-physiological concentrations of the bioactive form of 1,25D3, such that the ability of the differentiating cell to utilize physiologic concentrations of 25D3 has not been substantially investigated. Although controversy still exists on the normal concentrations of 25D, we used the Endocrine Society Clinical Practice Guidelines which define vitamin D deficiency as below 20ng/mL (50nM), insufficiency as 21–29 ng/mL (52.5nM-72.5nM), sufficient levels as more that 30ng/mL (75nM) [19]. In humans, 1,25D levels are regulated to be constant, such that the available level of 25D determines the amount of bioactive 1,25D that is generated in an activated MΦ and is therefore key to innate immune function [3]. Therefore, the aim of our work is to study the effects of physiological levels of 25D3 during IL-15 MΦ differentiation, function and antimicrobial response against *M*. *leprae*.

## Methods

### Statistical analysis

Experiments with three or more measurements were analyzed using One Way ANOVA or with Student-Newman-Keuls Method (*P<0.05, **P<0.01, ***P<0.005, ****P<0.001) for pairwise analyses using GraphPad Prism 7 software. Error bars represent the standard error of the mean between individual donor values. A two-tailed student’s t-test was used to compare two different experimental conditions.

### Ethics Statement

This study was conducted according to the principles expressed in the Declaration of Helsinki and was approved by the Institutional Review Board (IRB) of the University of California at Los Angeles (UCLA). Human peripheral blood from healthy donors was acquired with informed consent (UCLA Institutional Review Board #11–001927). All adult subjects provided written informed consent. Peripheral blood mononuclear cells (PBMCs) were isolated from the blood of healthy donors using Ficoll-Paque (GE healthcare) and monocytes were purified with plastic adherence as previously described [20].

### Macrophage differentiation

Adherent monocytes were cultured in the presence of IL-15 (R&D Systems, 200ng/ml) or IL-10 (R&D Systems, 10ng/ml) for 48 hours using Serum Free MΦ media (SFM) (Gibco) at 37°C and 5% CO_2_. Cell phenotypes were consistent with previously published data [10].

### Flow cytometry

The following antibody clones were used per manufacturers’ protocol for flow cytometry: CD209 (DCN46), CD163 (GHI/61), CD16 (3G8), CD14 (M5E2), and CAMP/LL37/FALL39/Cathelicidin Antibody (OSX12). Differentiated MΦwere harvested and stained as previously described [1, 4, 5, 20].

### Quantitative real-time PCR (qPCR)

RNA was harvested using TRIzol reagent (Life Technologies) via phenol-chloroform extraction, followed by RNA cleanup and DNase digestion using the RNeasy Miniprep Kit (Qiagen) as previously described [21]. cDNA was synthesized using iScript cDNA synthesis kit (Bio-Rad) and stored at -80°C. Primer sequences were used as follows: CYP27B1 F: ACC CGA CAC GGA GAC CTT C, CYP27B1 R: ATG GTC AAC AGC GTG GAC AC; CAMP F: TGG GCC TGG TGA TGC CT, CAMP R: CGA AGG ACA GCT TCC TTG TAG C H36B4 F: CCA CGC TGC TGA ACA TGC T, H36B4 R: TCG AAC ACC TGC TGG ATG AC. Real-time PCR was performed using SYBR Green (Kapa Biosystems) according to the manufacturers’ protocol. cDNA levels were normalized with H36B4 as the housekeeping gene. Relative CAMP mRNA levels were normalized to IL-15 MΦ differentiated in the absence of vitamin D and shown as fold-change (FC). Relative CYP27B1 mRNA levels were normalized to IL-15 MΦ baseline levels and shown as fold-change (FC) as previously described [1, 6, 10, 21].

### Cathelicidin protein levels in IL-15 MΦ (microscopy)

Primary human monocytes were seeded onto chamber slides (BD falcon) and treated with IL-15 with or without the presence of vitamin D. The cells were fixed and permeabilized using fixation/permeabilization solution kit (BD Bioscience) as indicated by the manufacturer. Cells were blocked with 10% human serum for 20 minutes and stained with CAMP/LL37/FALL39/Cathelicidin Antibody (OSX12) antibody at 10μg/mL overnight. The monolayers were washed three times with cold-PBS and stained with a biotinylated-horse anti-mouse antibody (Bio-Rad) at 10μg/mL at room temperature for one-hour. The monolayers were washed again three times with cold-PBS and stained with streptavidin-conjugated to Alexa Fluor 488 (Invitrogen) protected from light as previously described [1, 5]. The cells were washed with PBS and sealed with ProLong Gold antifade reagent with DAPI (Invitrogen). Microscopy images were analyzed with the SP8-SMD confocal microscope (Leica) at the Advanced Microscopy Laboratory Macro-Scale Imaging Laboratory, California Nanosystems Institute, UCLA [22].

### Cathelicidin protein levels in IL-15 MΦ (flow cytometry)

IL-15 MΦwere differentiated in the presence or absence of vitamin D in a 24-well tissue culture plate (Corning). The cells were harvested and fixed with 4% paraformaldehyde for 15 minutes at room temperature in a V-bottom plate (Corning). After fixation, the MΦ were permeabilized with 0.5% saponin (Sigma) in PBS and quickly washed with a series of PBS washes. The cells were then stained using same staining protocol as described above. The cells were acquired with a BD LSRII in the Janis V. Gorgi Flow Cytometry Core Laboratory at UCLA. All analysis was done using FlowJo 10.4.2 software.

### IL-15 MΦ phagocytosis (flow cytometry)

Cells were infected with PE-labeled *M*. *leprae* and harvested 24 hours post infection (PI). The cells were blocked with 10% human serum for 20 minutes at room temperature and stained with an anti-CD14 antibody as indicated by the manufacturer for 30 minutes on ice. The cells were then fixed with 4% paraformaldehyde for 15 minutes and analyzed by flow cytometry as previously described [4, 20]. The cells were acquired with a BD LSRII in the Janis V. Gorgi Flow Cytometry Core Laboratory at UCLA. All analysis was done using FlowJo 10.4.2 software. The same samples were also analyzed using IdEAS Software, ImageStream (Amnis) as explained below.

### ImageStream

The ImageStreamX MarkII imaging flow cytometer from Amnis Corporation was used for acquisition at 60X magnification. Anti-human CD14 antibody conjugated to PacBlue channel 1 was detected on the 405 nm laser at 30.00 mW, and PE labeled *M*. *leprae* channel 3 was detected off the 488 nm laser set at 70.00 mW. Acquisition was set to collect 5000 objects from the single cell population (Aspect Ratio Bright Field channel 4 vs. Area Bright Field channel 4).

Data was analyzed using the Amnis IDEAS software. A compensation matrix was first created using single-stained CD14 labeled macrophages and PE calibrite beads (BD). Compensated data was then applied to a template with a gating hierarchy. Focused cells were first selected from the higher population of the Gradient RMS histogram; single cells were then chosen using the same gating strategy applied during acquisition and lastly, PE and PacBlue double positive cells were then applied to the Spot Count Wizard. Two populations containing 10 or more cells, each expressing high or low values of spots (single PE labeled *M*. *leprae* bacterium) were manually selected. The Spot Count Wizard has the ability to measure uptake and count spots, thus providing individual bacterial counts per cell.

### *M*. *leprae* and the assay of antimicrobial assay activity

IL-15 MΦwere differentiated with or without the indicated amount of 25D for 48 hours. The MΦ were then infected with *M*. *leprae* overnight at an MOI of 10. The cells were washed with SFM to remove extracellular bacteria and treated with IL-15 and the indicated amounts of 25D. The infection progressed for 24 hours, 48 hours and 120 hours and all the material in the well was harvested into a 15mL conical tube. To ensure that all the material in the well was harvested, each well was washed with a series of cold PBS-EDTA washes and accumulated into their respective 15mL conical tube. Each 15mL conical tube was placed into a centrifuge for 300xg for 10 mins at 4°C. The supernatants were carefully removed and the genomic DNA was isolated as previously described [21, 23]. We compared RLEP DNA levels of *M*. *leprae* with the H36B4 levels of the IL-15 MΦto measure bacterial burden using real-time PCR using SYBR Green as indicated by the manufacturer (Kapa Biosystems) [24, 25]. The following primers sequences were used: RLEP F: GCA GCA GTA TCG TGT TAG TGA A, RLEP R: CGC TAG AAG GTT GCC GTA T; H36B4 F: CCA CGC TGC TGA ACA TGC T, H36B4 R: TCG AAC ACC TGC TGG ATG AC. The bacteria burden at each time point was normalized to IL-15 MΦdifferentiated without vitamin D to quantify relative bacteria burden.

## Results

### MΦ phenotype is sustained in SFM independent of 25D3 status

To investigate the effect of 25D3 on MΦ differentiation, we used SFM, allowing us to control the amount of 25D3 in the culture. SFM contains neither 25D3 nor any other vitamin D analogues, such that 25D3 can be added at defined concentrations. Thus, SFM has an advantage over fetal calf sera or human sera that have varying amounts of 25D3. Previously, IL-15 and IL-10 were shown to induce the differentiation of monocytes into distinct MΦ populations, however, these experiments were performed using FCS, which contains low levels of 25D3. Thus, it was unclear whether vitamin D status affects the differentiation of monocytes into IL-15 MΦ and IL-10 MΦ.

**Fig 1:**
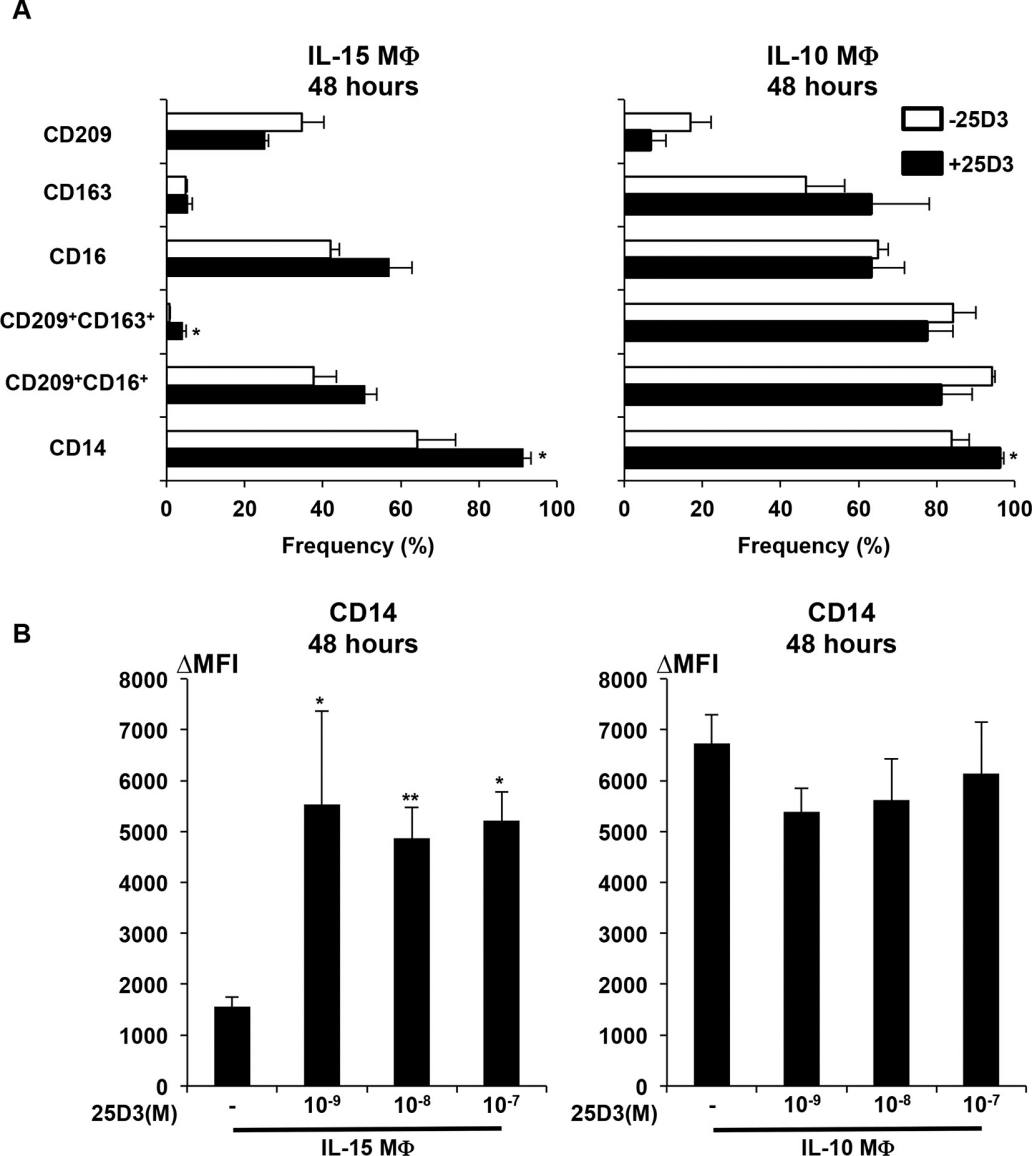
Phenotype of IL-15 MΦ and IL-10 MΦ differentiated in 25D3. **A)** IL-15 MΦ (left) or IL-10 MΦ (right) were differentiated with or without the presence of 10^-8^M 25D3 for 48 hours. Cells were stained with indicated markers and analyzed by flow cytometry. Data is represented as average frequency (%) +/- SEM relative to isotype control (n = 3–4). **B)** Data is represented as the change in mean fluorescence intensity of CD14 (CD14 ΔMFI) +/- SEM relative to isotype control (n = 4). *P<0.05, **P<0.01.

All experiments here involve MΦs derived from cytokine treated monocytes as previously reported [10]. Monocytes were cultured with either IL-15 or IL-10 for 48 hours in SFM with or without the addition of 25D3 (10^-8^M 25D3). This is equivalent to the physiologic concentration in vitamin D sufficient serum of 10^-7^M 25D3, which is then diluted to 10% serum in cell cultures [1, 26]. Both IL-15 and IL-10 induced CD209 expression as assessed by flow cytometry, but only IL-10 induced CD163 expression (Fig 1A), similar to differentiation in FCS [10]. Examining co-expression of CD209 and CD163, we found that IL-15 induced CD209^+^CD163^-^ MΦ, whereas IL-10 induced CD209^+^CD163^+^ MΦ, accounting ~80% of cells. We also found that the IL-15 MΦand IL-10 MΦ derived in SFM express the MΦ specific marker CD16. The average surface expression of CD16 increased in IL-15 MΦ when differentiated in 25D3 to similar levels seen on IL-10 MΦ, but was not significant (p = 0.07). In addition, we observed that 25D3 status did not significantly alter the surface expression of CD209, CD163, CD16 or the coexpression of CD209^+^CD16^+^ whether derived using IL-15 or IL-10 (Fig 1A). The frequency of CD14 was expressed on IL-15 MΦ, with a small but significant enhancement by 25D3, to the level expressed on IL-10 MΦ (Fig 1A). This was also reflected in an increase in cellular abundance as measured by the change in mean fluorescence intensity (ΔMFI) for MΦ derived in IL-15 but not IL-10 (Fig 1B). Overall, SFM supported the differentiation of monocytes by IL-15 and IL-10 into divergent MΦ phenotypes, which were generally similar whether differentiated in the presence or absence of 25D3.

### 25D3 status triggers vitamin D-dependent antimicrobial profile in IL-15 MΦ

**Fig 2:**
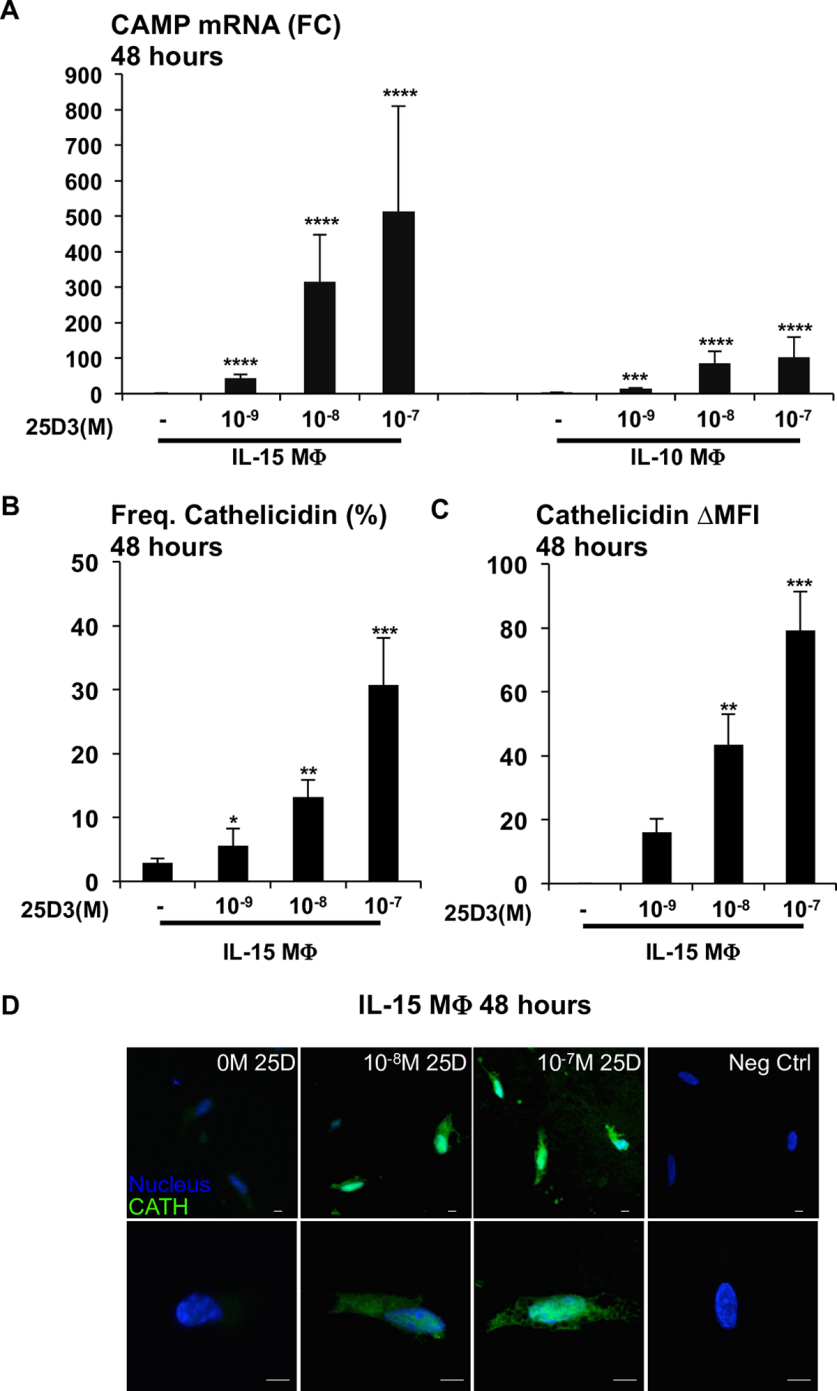
CAMP mRNA and cathelicidin protein expression in IL-15 MΦ is dependent on 25D3 status. **A)** IL-15 MΦand IL-10 MΦ were differentiated in increasing concentrations of 25D3 (0 to 10^-7^M) for 48 hours and CAMP mRNA levels were determined by qPCR. Fold change was calculated relative to IL-15 MΦ differentiated in no 25D3. Data is represented as average fold change +/- SEM (n = 4–7). **B)** The frequency (%) of cathelicidin protein expression of IL-15 MΦ differentiated in increasing concentrations of 25D3 (0 to 10^-7^M) for 48 hours. Data is represented as average frequency +/- SEM (n = 6) relative to isotype control. **C)** The change in cathelicidin mean fluorescence intensity (cathelicidin ΔMFI) in IL-15 MΦ differentiated in increasing concentrations of 25D3 (0 to 10^-7^M) for 48 hours. The data is represented as the average cathelicidin ΔMFI +/- SEM (n = 6) relative to no 25D3. **D)** Monocytes were treated with IL-15 in the presence of increasing concentrations of 25D3 (0 to 10^-7^M) for 48 hours. Cells were harvested and stained with either an anti-cathelicidin antibody or IgG1 isotype control antibody and analyzed by confocal microscopy. (Original magnification, 400x (top row)); (6.5x zoom of original magnification (bottom row)). Scale bar = 5μm. (n = 3) *P<0.05, **P<0.01, ***P<0.005, ****P<0.001.

Although 25D3 did not dramatically affect the differentiation of MΦ by phenotype, we next investigated whether the presence of 25D3 during differentiation affected MΦ function. The induction of the antimicrobial protein cathelicidin is essential for the vitamin D-dependent antimicrobial response against intracellular mycobacteria in infected MΦ [5, 27]. To determine whether 25D3 status during MΦ differentiation results in activation of the vitamin D-dependent antimicrobial pathway, we treated monocytes with IL-15 and IL-10 in the presence of increasing concentrations of 25D3 during differentiation and measured CAMP mRNA levels after 48 hours by qPCR. Conditioning during differentiation of both IL-15 MΦ and IL-10 MΦ in SFM supplemented with increasing level of 25D3 resulted in a significant dose-dependent induction of CAMP mRNA (Fig 2A). At all concentrations of 25D3, the CAMP mRNA expression was more robust in IL-15 MΦ relative to IL-10 MΦ. We observed a ~315-fold induction of CAMP mRNA in SFM supplemented with 10^-8^M 25D3 as compared to media without 25D3 in IL-15 MΦ, and ~500-fold induction of CAMP mRNA in SFM containing 10^-7^M 25D3 (Fig 2A). In comparison, we observed a ~80-fold induction of CAMP mRNA in SFM containing 10^-8^M 25D3 in IL-10 MΦ and a ~100-fold induction of CAMP mRNA in SFM containing 10^-7^M 25D3 (Fig 2A). The baseline values of CYP27B1 mRNA expression was not significant between IL-15 MΦand IL-10 MΦ(S1 Fig).

We determined whether the induction of CAMP mRNA was associated with expression of cathelicidin protein using intracellular flow cytometry. The CAMP mRNA expression levels in IL-15 MΦ correlated with both the frequency of cathelicidin and the cathelicidin protein abundance as measured by ΔMFI (Fig 2B and 2C). The average frequency of cathelicidin was ~13% in IL-15 MΦ derived in 10^-8^M 25D3 and ~30% in 10^-7^M 25D3 supplemented SFM (Fig 2B). The ΔMFI was ~45 AU in IL-15 MΦ derived in 10^-7^M 25D3 and ~80 AU in 10^-8^M 25D3 supplemented SFM (Fig 2C).

Representative fluorescence microscopy images of IL-15 MΦ conditioned in 25D3 indicates that cathelicidin protein accumulates in the intracellular vesicles proximal to the host nucleus, but not in IL-15 MΦdifferentiated in no 25D3 (Fig 2D). These data collectively indicate that cathelicidin mRNA and protein expression directly correlate with 25D3 status during IL-15 induced MΦ differentiation.

### Vitamin D status does not alter IL15-MΦphagocytic function

**Fig 3:**
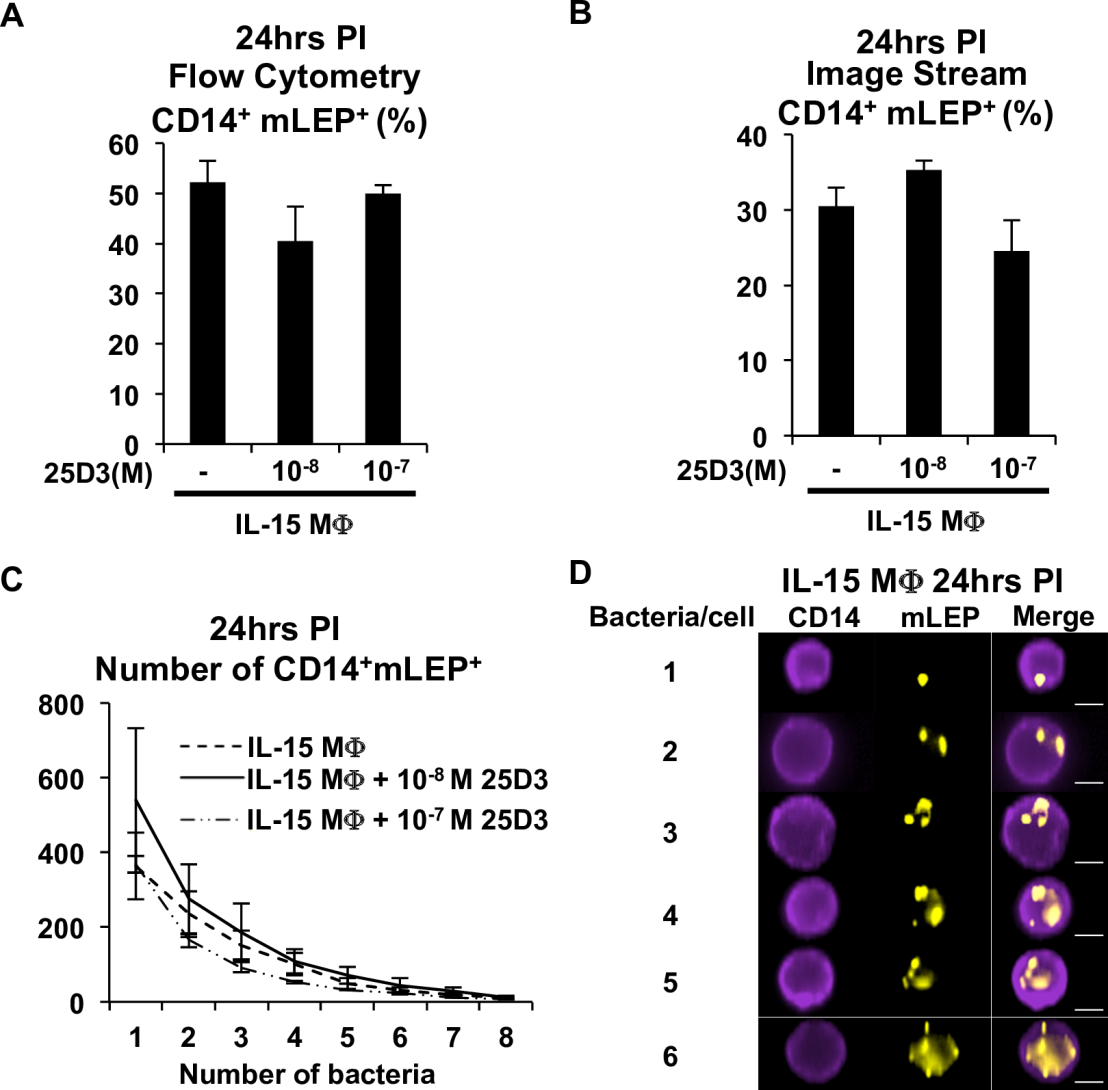
IL-15 MΦ phagocytose *M*. *leprae*. IL-15 M were differentiat;ed in the presence of increasing concentrations of 25D3 (0 to 10^-7^M) for 48 hours and infected with PE-labeled *M*. *leprae* (mLEP) for 24 hours and analyzed with either **A)** flow cytometry or **B)** image stream flow cytometry. Data is represented as average frequency of IL-15 MΦ that are positive for both CD14 and mLEP +/- SEM relative to uninfected and isotype controls (n = 3). **C)** The number of CD14^+^ IL-15 MΦ that contained 1–8 bacteria was analyzed with an unsupervised spot counting function Ideas software. Data is represented as an average number of CD14^+^mLEP^+^ for each bacterium count +/- SEM (n = 3). **D)** Representative microscopy images from image stream flow cytometry analysis (1–6 bacteria per cell). Scale bar = 7μm. (n = 3).

An important function of antimicrobial MΦ is the phagocytosis of pathogens to contain microbes in the host *in vivo*. However, it is unclear how vitamin D status may alter the phagocytic function of the MΦ during *M*. *leprae* infection. To assess phagocytic function, IL-15 MΦwere conditioned with or without 25D3, infected with PE labeled-*M*. *leprae* for 24 hours, stained for the MΦ specific marker CD14 and phagocytosis was analyzed by flow cytometry and image stream flow cytometry. The efficiency of *M*. *leprae* infection in IL-15 MΦdifferentiated in the absence of vitamin D or in the presence of either 10^-8^M 25D3 or 10^-7^M 25D3, was not statistically different, although somewhat greater in culture in which no 25D3 was present (Fig 3A). Image stream flow cytometry analysis of the same samples demonstrated a frequency of infection of CD14^+^mLEP^+^ cells ranging from 30%, 35%, to 25%, when conditioned with no vitamin D, 10^-8^M 25D3 or 10^-7^M 25D3, respectively (Fig 3B). Using an unsupervised spot counting function of image stream flow cytometry, we determined the frequency of CD14^+^ cells containing varying numbers of intracellular *M*. *leprae* and no effect of 25D3 was observed (Fig 3C). Images from image flow cytometry analysis show the number of bacteria per MΦ (Fig 3D). Cells that contained either 7 or 8 bacteria all showed large clumps of bacteria in which were difficult to interpret as an accurate number of bacteria. Overall no significant difference was observed in the number of bacteria per cell. These data indicate that vitamin D status does not alter the phagocytic capacity of IL-15 MΦ.

### Vitamin D status in IL-15 MΦ triggers antimicrobial response against *M*. *leprae*

After the phagocytosis of invading pathogens, a major function of MΦ is to effectively mount an antimicrobial response to defend the host. However, it is unclear whether sufficient levels of 25D3 will provide MΦ with the capacity to mount an antimicrobial response. To investigate whether 25D3 status during MΦ differentiation affects the antimicrobial response, we simultaneously measured the kinetics of CAMP mRNA induction and antimicrobial activity against *M*. *leprae* in IL-15 MΦ. IL-15 MΦ were conditioned with or without 25D3, infected with *M*. *leprae* for 24, 48, and 120 hours, at which time both RNA and DNA were harvested.

**Fig 4:**
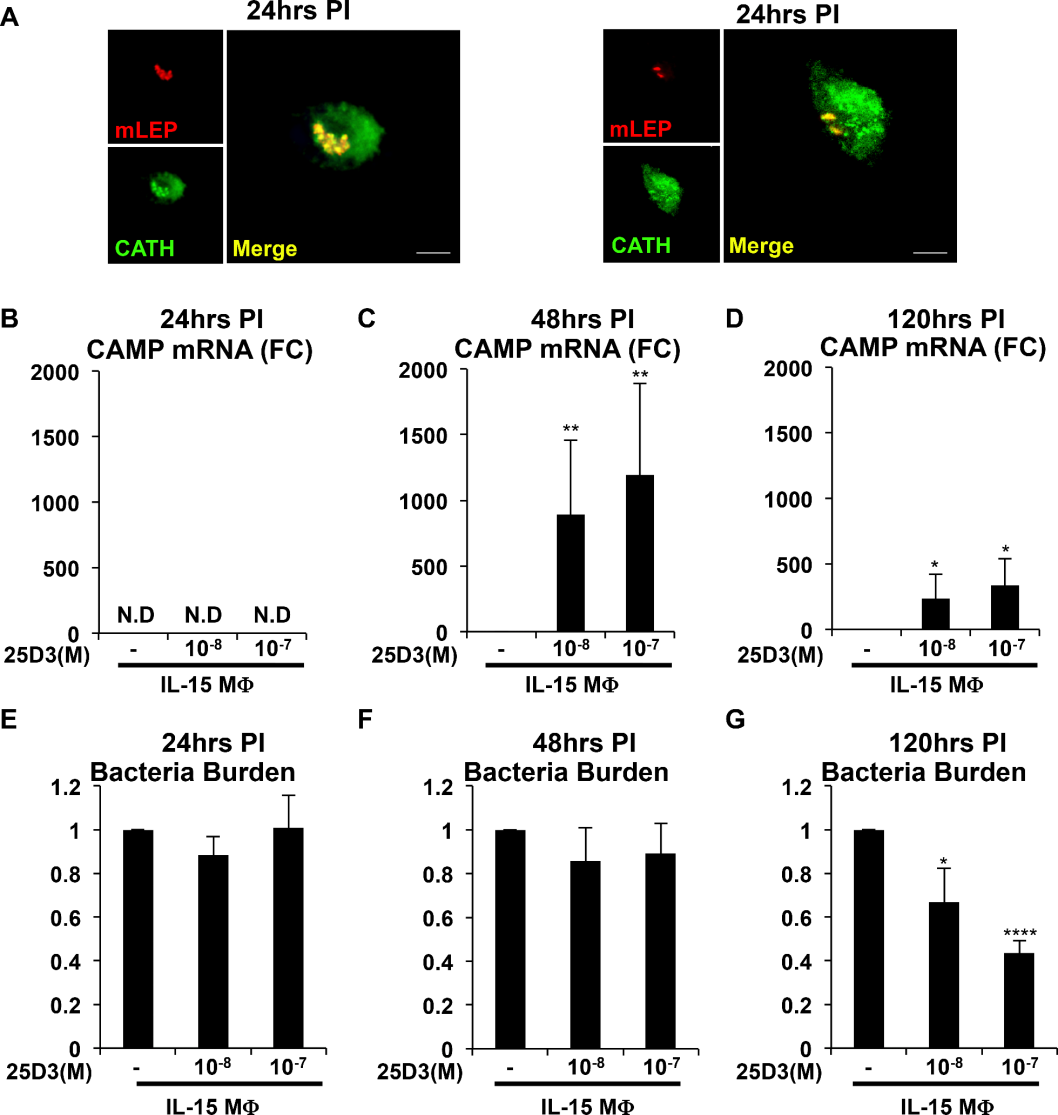
25D3 status contributes to IL-15 MΦ antimicrobial response. **A)** Colocalization of *M*. *leprae* (mLEP = red) and cathelicidin (CATH = green) 24 hours PI in IL-15 MΦdifferentiated in the presence of 25D3 for 48 hours. (Original magnification, 400); (6.5x zoom of original magnification). Scale bar = 5μm. IL-15 MΦ were differentiated in increasing concentrations of 25D3 (0 to 10^-7^M) for 48 hours and infected with *M*. *leprae* for **B)** 24 hours (n = 3), **C)** 48 hours (n = 5) or **D)** 120 hours (n = 5) and CAMP mRNA expression was assessed by qPCR. Data is represented as the average fold change in CAMP mRNA +/- SEM relative to IL-15 MΦ differentiated in no 25D3. IL-15 MΦ were differentiated in increasing concentrations of 25D3 (0 to 10^-7^M) for 48 hours and infected with *M*. *leprae* for **E)** 24 hours (n = 3), **F)** 48 hours (n = 5) or **G)** 120 hours (n = 5) and bacteria burden was assessed by qPCR. Data is represented as the average change in bacteria burden +/- SEM relative to IL-15 MΦ differentiated in no 25D3. *P<0.05, **P<0.01, ***P<0.005, ****P<0.001.

The levels of CAMP mRNA in *M*. *leprae* infected IL-15 MΦ at 24 hours were relatively low as compared to the previous experiments in which CAMP mRNA was measured in uninfected MΦ, to the extent that CAMP mRNA was not detectable in some donors at this time point. However, the cathelicidin protein colocalized with *M*. *leprae* in IL-15 MΦdifferentiated in 25D3, but not in IL-15 MΦdifferentiated in no 25D3 24 hours post *M*. *leprae* infection (Fig 4A). The low level of CAMP mRNA expression was not different whether the MΦ were differentiated in the presence or absence of 25D3 (Fig 4B). One possibility for the absence of CAMP mRNA but presence of cathelicidin protein at 24 hrs post infection is that upon infection with *M*. *leprae* the CAMP mRNA is downregulated yet the protein was already synthesized during differentiation.

At 48 hours after *M*. *leprae* infection, CAMP mRNA expression was approximately 1000 fold in the IL-15 MΦ differentiated in 25D3, at either 10^-8^M 25D3 or 10^-7^M 25D3 (Fig 4C). At 120-hours post infection, the relative CAMP mRNA remained significantly increased in the MΦ differentiated in 25D3, approximately 200–330 fold greater than in MΦ differentiated in the absence of 25D3 (Fig 4D).

The *M*. *leprae* burden was measured in the infected IL-15 MΦ according to the level of bacterial DNA. The *M*. *leprae* burden in infected IL-15 MΦ was not affected by the presence of 25D3 during differentiation as assessed at 24 or 48 hours (Fig 4E and 4F). In one of the five donors, we noted a reduction in bacterial burden at 48 hours. However at 120 hours post infection the relative bacteria burden significantly decreased to 0.67 and 0.44 in IL-15 MΦ differentiated in 10^-8^M and 10^-7^M 25D3 compared to no 25D3, respectively (Fig 4G). The decrease in viability of *M*. *leprae* at 120 hours was not due to differences in macrophage number, as H36B4 levels remained constant. Antimicrobial activity against *M*. *leprae* was detected in IL-15 MΦ differentiated in 25D3 in all five donors. These data indicate that the presence of 25D3 during the IL-15 MΦ differentiation program and throughout *M*. *leprae* infection contributes to the vitamin D-dependent antimicrobial response against by *M*. *leprae*.

## Discussion

The ability of human MΦ to mount an effective response against intracellular mycobacteria depends in part upon their ability to induce the vitamin D-dependent antimicrobial pathway [1, 2, 5, 28]. Although sufficient levels of vitamin D are required for optimal MΦ effector function, previous studies have indicated that myeloid cell differentiation and function can be altered by vitamin D bioavailability. Here, we investigated whether the level of 25D influences MΦ differentiation and programming of an antimicrobial response against *M*. *leprae*. The distinct phenotypes of IL-15 MΦ and IL-10 MΦ were largely sustained during differentiation from monocytes regardless of 25D3 status, yet onlyIL-15 MΦdifferentiated in the presence 25D3 robustly triggered the expression of CAMP mRNA and cathelicidin protein levels in a dose-dependent manner. Vitamin D status did not alter the phagocytic function of IL-15 MΦ, but a significant decrease in bacteria burden against *M*. *leprae* was observed at 120 hours post-infection. These data indicate that 25D3 status during IL-15 MΦdifferentiation permits the induction of an antimicrobial response against intracellular *M*. *leprae*.

It is important for the host to mount an antimicrobial response against intracellular mycobacteria before the bacteria employ evasion mechanisms that help establish infection and progress to clinical disease [22, 23]. A key finding of the present study was that the addition of 25D3 during the IL-15 induced differentiation of monocytes into MΦ led to a robust induction of the vitamin D-dependent antimicrobial pathway, including the induction of cathelicidin and an antimicrobial response against *M*. *leprae*. We detected a 315-fold induction of CAMP mRNA in IL-15 MΦ differentiated in the presence 10^-8^M 25D3 as compared to SFM without 25D3 and a 1/3 reduction in the *M*. *leprae* burden in infected cells. Previously we have shown that IL-15 MΦ supplemented with 10^-8^M 25D3 post-differentiation demonstrated a 5-fold increase in CAMP mRNA and a ~50% reduction in avirulent *M*. *tuberculosis* (H37ra) viability [4]. However, these IL-15 MΦ were differentiated in 10% 25D insufficient FCS (16nM). These data collectively suggest that 25D levels during IL-15 MΦ differentiation facilitate their antimicrobial function as part of the innate immune response. There are situations that allow the pathogen to escape the vitamin D antimicrobial response. For example, genetic polymorphisms in the VDR have been associated with increased susceptibility to mycobacterial infection [29]. *M*. *leprae* evades the vitamin D antimicrobial response via the induction of a microRNA that targets the pathway [23], and by induction of type 1 interferon leading to IL-10 and subsequent suppression of the vitamin D pathway [22]. These data imply that upon the onset of microbial challenge, monocytes that are recruited to the site of infection are dependent on the presence of sufficient levels of 25D to differentiate into powerful IL-15 MΦthat fend of *M*. *leprae* evasion mechanisms and effectively reduce bacterial viability [1, 5, 30].

The addition of 25D3 during the IL-15 induced differentiation of monocytes into MΦ affected antimicrobial function, but we observed little change in cell phenotype. Regardless if the MΦ were differentiated with or without 25D3, we found that the distinct phenotypes of IL-15 MΦ and IL-10 MΦ were largely not affected. In particular, the IL-15 MΦ were CD209^+^CD163^-^ and the IL-10 MΦ were CD209^+^CD163^+^. Only IL-15 MΦ differentiated in 25D3 demonstrated both a significant increase in CD14 frequency and cellular abundance. Although CD14 is a marker that identifies VDR-activated MΦs [31], we have no evidence that the differences in CD14 expression directly affected function as phagocytic capacity was not affected. In contrast to our findings, the addition of 1,25D3 during differentiation of monocytes into MΦ by macrophage colony-stimulating factor (M-CSF) decreased phagocytic function and the release of pro-inflammatory cytokines [18]. Similarly, the addition of 25D3 or 1,25D3 during differentiation of monocytes into dendritic cells by granulocyte-macrophage colony-stimulating factor (GM-CSF) plus IL-4 decreased the expression of DC-specific surface markers CD1a, CD80, CD86, and MHC class-II, as well as antigen presentation capacity [16, 17]. In these experiments the levels of 1,25D3 were supraphysiologic, although 25D3 was added at physiologic levels. Taken together with our findings, these findings suggest that although physiologic levels of 25D may alter the differentiation of DC, it permits MΦ differentiation and enhances MΦ antimicrobial function.

In the present study we determined that clinically sufficient levels of 25D3 led to a functional difference in IL-15 MΦ, relative to MΦ differentiated in the absence of 25D3. In humans, there is a range of 25D levels that can be classified from deficient (45nM) to sufficient (98nM) [2]. Previously, we compared the ability of African American sera and Caucasian sera to induce the expression of the mRNAs encoding the antimicrobial peptides cathelicidin and beta-defensin 2 and found that African American sera was less capable to induce the antimicrobial peptides *ex vivo* due to the relatively lower 25D sera levels [1, 2]. Both exogenous 25D supplementation to African American sera *ex vivo* and 25D supplementation to vitamin D deficient individuals *in vivo* significantly enhanced CAMP mRNA expression in activated monocytes and MΦ *in vitro* [1, 2, 32].

Our data suggest that people with higher levels of vitamin D will derive MΦ with more antimicrobial function that could prevent the establishment of infection; however, testing the effects of vitamin D status on the prevention of infection by *M*. *tuberculosis* is challenging. The ability to acquire a large enough population with differential 25D levels randomly will be difficult as 25D status strongly correlates with season [33], as such there are few studies that investigate the interaction of 25D status with infection by the pathogen. Deficient levels of 25D in household contacts of TB patients demonstrated either increased latent TB incidence or positive tuberculoid skin tests [33, 34]; however the number of patients that acquire active TB is unclear [35–37]. These data support continued and more thorough investigation into whether vitamin D supplementation of deficient and insufficient individuals *in vivo* can enhance the MΦ antimicrobial response against mycobacterial infections and contain the spread and outcome of disease.

In conclusion, we found that vitamin D-dependent antimicrobial MΦ differentiated in the presence of sufficient levels of 25D3 sustain a MΦ phenotype and exhibit an antimicrobial response against *M*. *leprae*. Our model indicates that vitamin D-dependent antimicrobial MΦ differentiated in the presence of sufficient 25D are capable of intrinsic microbicidal activity against infection. In contrast, the same MΦ differentiated in the low levels of 25D require the addition of exogenous 25D to induce activity [1, 2, 5, 32]. These data suggest that sufficient levels of 25D at the site of microbial infection allow recruited monocytes to differentiate into vitamin D-dependent antimicrobial MΦ with the capacity to effectively reduce the viability of intracellular bacteria. Future clinical trials that study the relationship between vitamin D supplementation and susceptibility to microbial infection will determine if the prophylactic effects of vitamin D are therapeutically beneficial.

## Supporting information

S1 Fig CYP27B1 mRNA expression in IL-15 MΦ and IL-10 MΦ. Primary human monocytes were treated with either IL-15 or IL-10 for 48 hours in SFM. No significant difference is observed between CYP27B1 mRNA expression levels between IL-15 MΦ and IL-10 MΦ.
(TIF)

## References

[1] Liu PT, Stenger S, Li H, Wenzel L, Tan BH, Krutzik SR, et al Toll-like receptor triggering of a vitamin D-mediated human antimicrobial response. Science. 2006;311(5768):1770–3. 10.1126/science.1123933 . 16497887

[2] Fabri M, Stenger S, Shin DM, Yuk JM, Liu PT, Realegeno S, et al Vitamin D is required for IFN-gamma-mediated antimicrobial activity of human macrophages. Sci Transl Med. 2011;3(104):104ra2 10.1126/scitranslmed.3003045 ; PubMed Central PMCID: PMC3269210. 21998409 PMC3269210

[3] Fabri M, Modlin RL. A vitamin for autophagy. Cell Host Microbe. 2009;6(3):201–3. 10.1016/j.chom.2009.08.008 . 19748462

[4] Krutzik SR, Hewison M, Liu PT, Robles JA, Stenger S, Adams JS, et al IL-15 links TLR2/1-induced macrophage differentiation to the vitamin D-dependent antimicrobial pathway. J Immunol. 2008;181(10):7115–20. ; PubMed Central PMCID: PMCPMC2678236. 18981132 10.4049/jimmunol.181.10.7115 PMC2678236

[5] Liu PT, Stenger S, Tang DH, Modlin RL. Cutting edge: vitamin D-mediated human antimicrobial activity against Mycobacterium tuberculosis is dependent on the induction of cathelicidin. J Immunol. 2007;179(4):2060–3. . 17675463 10.4049/jimmunol.179.4.2060

[6] Montoya D, Inkeles MS, Liu PT, Realegeno S, Teles RM, Vaidya P, et al IL-32 is a molecular marker of a host defense network in human tuberculosis. Sci Transl Med. 2014;6(250):250ra114 10.1126/scitranslmed.3009546 ; PubMed Central PMCID: PMCPMC4175914. 25143364 PMC4175914

[7] White JH. Vitamin D signaling, infectious diseases, and regulation of innate immunity. Infect Immun. 2008;76(9):3837–43. 10.1128/IAI.00353-08 ; PubMed Central PMCID: PMCPMC2519414. 18505808 PMC2519414

[8] White JH. Vitamin D as an inducer of cathelicidin antimicrobial peptide expression: past, present and future. J Steroid Biochem Mol Biol. 2010;121(1–2):234–8. 10.1016/j.jsbmb.2010.03.034 . 20302931

[9] Bursuker I, Goldman R. On the origin of macrophage heterogeneity: a hypothesis. J Reticuloendothel Soc. 1983;33(3):207–20. . 6300396

[10] Montoya D, Cruz D, Teles RM, Lee DJ, Ochoa MT, Krutzik SR, et al Divergence of macrophage phagocytic and antimicrobial programs in leprosy. Cell Host Microbe. 2009;6(4):343–53. 10.1016/j.chom.2009.09.002 ; PubMed Central PMCID: PMCPMC2764558. 19837374 PMC2764558

[11] Koeffler HP, Amatruda T, Ikekawa N, Kobayashi Y, DeLuca HF. Induction of macrophage differentiation of human normal and leukemic myeloid stem cells by 1,25-dihydroxyvitamin D3 and its fluorinated analogues. Cancer Res. 1984;44(12 Pt 1):5624–8. . 6594194

[12] Taimi M, Chateau MT, Cabane S, Marti J. Synergistic effect of retinoic acid and 1,25-dihydroxyvitamin D3 on the differentiation of the human monocytic cell line U937. Leuk Res. 1991;15(12):1145–52. . 1766263 10.1016/0145-2126(91)90183-t

[13] Jensen SS, Madsen MW, Lukas J, Binderup L, Bartek J. Inhibitory effects of 1alpha,25-dihydroxyvitamin D(3) on the G(1)-S phase-controlling machinery. Mol Endocrinol. 2001;15(8):1370–80. 10.1210/mend.15.8.0673 . 11463860

[14] Studzinski GP, Garay E, Patel R, Zhang J, Wang X. Vitamin D receptor signaling of monocytic differentiation in human leukemia cells: role of MAPK pathways in transcription factor activation. Curr Top Med Chem. 2006;6(12):1267–71. . 16848740 10.2174/156802606777864935

[15] Samuel S, Sitrin MD. Vitamin D's role in cell proliferation and differentiation. Nutr Rev. 2008;66(10 Suppl 2):S116–24. 10.1111/j.1753-4887.2008.00094.x . 18844838

[16] Hewison M, Freeman L, Hughes SV, Evans KN, Bland R, Eliopoulos AG, et al Differential regulation of vitamin D receptor and its ligand in human monocyte-derived dendritic cells. J Immunol. 2003;170(11):5382–90. . 12759412 10.4049/jimmunol.170.11.5382

[17] Piemonti L, Monti P, Sironi M, Fraticelli P, Leone BE, Dal Cin E, et al Vitamin D3 affects differentiation, maturation, and function of human monocyte-derived dendritic cells. J Immunol. 2000;164(9):4443–51. . 10779743 10.4049/jimmunol.164.9.4443

[18] Arboleda Alzate JF, Rodenhuis-Zybert IA, Hernandez JC, Smit JM, Urcuqui-Inchima S. Human macrophages differentiated in the presence of vitamin D3 restrict dengue virus infection and innate responses by downregulating mannose receptor expression. PLoS Negl Trop Dis. 2017;11(10):e0005904 10.1371/journal.pntd.0005904 ; PubMed Central PMCID: PMCPMC5653353. 29020083 PMC5653353

[19] Holick MF, Binkley NC, Bischoff-Ferrari HA, Gordon CM, Hanley DA, Heaney RP, et al Evaluation, treatment, and prevention of vitamin D deficiency: an Endocrine Society clinical practice guideline. J Clin Endocrinol Metab. 2011;96(7):1911–30. 10.1210/jc.2011-0385 . 21646368

[20] Krutzik SR, Tan B, Li H, Ochoa MT, Liu PT, Sharfstein SE, et al TLR activation triggers the rapid differentiation of monocytes into macrophages and dendritic cells. Nat Med. 2005;11(6):653–60. 10.1038/nm1246 ; PubMed Central PMCID: PMC1409736. 15880118 PMC1409736

[21] Wheelwright M, Kim EW, Inkeles MS, De Leon A, Pellegrini M, Krutzik SR, et al All-trans retinoic acid-triggered antimicrobial activity against Mycobacterium tuberculosis is dependent on NPC2. J Immunol. 2014;192(5):2280–90. 10.4049/jimmunol.1301686 ; PubMed Central PMCID: PMCPMC3954114. 24501203 PMC3954114

[22] Teles RM, Graeber TG, Krutzik SR, Montoya D, Schenk M, Lee DJ, et al Type I interferon suppresses type II interferon-triggered human anti-mycobacterial responses. Science. 2013;339(6126):1448–53. 10.1126/science.1233665 ; PubMed Central PMCID: PMC3653587. 23449998 PMC3653587

[23] Liu PT, Wheelwright M, Teles R, Komisopoulou E, Edfeldt K, Ferguson B, et al MicroRNA-21 targets the vitamin D-dependent antimicrobial pathway in leprosy. Nat Med. 2012;18(2):267–73. 10.1038/nm.2584 ; PubMed Central PMCID: PMCPMC3274599. 22286305 PMC3274599

[24] Kim EW, Nadipuram SM, Tetlow AL, Barshop WD, Liu PT, Wohlschlegel JA, et al The Rhoptry Pseudokinase ROP54 Modulates Toxoplasma gondii Virulence and Host GBP2 Loading. mSphere. 2016;1(2). 10.1128/mSphere.00045-16 ; PubMed Central PMCID: PMC4863586. 27303719 PMC4863586

[25] Martinez AN, Lahiri R, Pittman TL, Scollard D, Truman R, Moraes MO, et al Molecular determination of Mycobacterium leprae viability by use of real-time PCR. J Clin Microbiol. 2009;47(7):2124–30. 10.1128/JCM.00512-09 ; PubMed Central PMCID: PMCPMC2708532. 19439537 PMC2708532

[26] Bruns H, Buttner M, Fabri M, Mougiakakos D, Bittenbring JT, Hoffmann MH, et al Vitamin D-dependent induction of cathelicidin in human macrophages results in cytotoxicity against high-grade B cell lymphoma. Sci Transl Med. 2015;7(282):282ra47 10.1126/scitranslmed.aaa3230 . 25855493

[27] Adams JS, Hewison M. Update in vitamin D. J Clin Endocrinol Metab. 2010;95(2):471–8. 10.1210/jc.2009-1773 ; PubMed Central PMCID: PMCPMC2840860. 20133466 PMC2840860

[28] Martineau AR, Wilkinson KA, Newton SM, Floto RA, Norman AW, Skolimowska K, et al IFN-gamma- and TNF-independent vitamin D-inducible human suppression of mycobacteria: the role of cathelicidin LL-37. J Immunol. 2007;178(11):7190–8. . 17513768 10.4049/jimmunol.178.11.7190

[29] Bornman L, Campbell SJ, Fielding K, Bah B, Sillah J, Gustafson P, et al Vitamin D receptor polymorphisms and susceptibility to tuberculosis in West Africa: a case-control and family study. J Infect Dis. 2004;190(9):1631–41. 10.1086/424462 . 15478069

[30] Liu PT, Schenk M, Walker VP, Dempsey PW, Kanchanapoomi M, Wheelwright M, et al Convergence of IL-1beta and VDR activation pathways in human TLR2/1-induced antimicrobial responses. PLoS One. 2009;4(6):e5810 10.1371/journal.pone.0005810 ; PubMed Central PMCID: PMCPMC2686169. 19503839 PMC2686169

[31] Zamani F, Zare Shahneh F, Aghebati-Maleki L, Baradaran B. Induction of CD14 Expression and Differentiation to Monocytes or Mature Macrophages in Promyelocytic Cell Lines: New Approach. Adv Pharm Bull. 2013;3(2):329–32. 10.5681/apb.2013.053 ; PubMed Central PMCID: PMCPMC3848216. 24312856 PMC3848216

[32] Adams JS, Ren S, Liu PT, Chun RF, Lagishetty V, Gombart AF, et al Vitamin d-directed rheostatic regulation of monocyte antibacterial responses. J Immunol. 2009;182(7):4289–95. 10.4049/jimmunol.0803736 ; PubMed Central PMCID: PMCPMC2683618. 19299728 PMC2683618

[33] Balcells ME, Garcia P, Tiznado C, Villarroel L, Scioscia N, Carvajal C, et al Association of vitamin D deficiency, season of the year, and latent tuberculosis infection among household contacts. PLoS One. 2017;12(4):e0175400 10.1371/journal.pone.0175400 ; PubMed Central PMCID: PMCPMC5389794. 28403225 PMC5389794

[34] Ganmaa D, Giovannucci E, Bloom BR, Fawzi W, Burr W, Batbaatar D, et al Vitamin D, tuberculin skin test conversion, and latent tuberculosis in Mongolian school-age children: a randomized, double-blind, placebo-controlled feasibility trial. Am J Clin Nutr. 2012;96(2):391–6. 10.3945/ajcn.112.034967 ; PubMed Central PMCID: PMCPMC3396446. 22760564 PMC3396446

[35] LoBue PA, Castro KG. Is it time to replace the tuberculin skin test with a blood test? JAMA. 2012;308(3):241–2. 10.1001/jama.2012.7511 ; PubMed Central PMCID: PMCPMC4629850. 22797639 PMC4629850

[36] Esmail H, Barry CE 3rd, Young DB, Wilkinson RJ. The ongoing challenge of latent tuberculosis. Philos Trans R Soc Lond B Biol Sci. 2014;369(1645):20130437 10.1098/rstb.2013.0437 ; PubMed Central PMCID: PMCPMC4024230. 24821923 PMC4024230

[37] Hornum M, Mortensen KL, Kamper AL, Andersen AB. Limitations of the QuantiFERON-TB Gold test in detecting Mycobacterium tuberculosis infection in immunocompromised patients. Eur J Intern Med. 2008;19(2):137–9. 10.1016/j.ejim.2007.03.020 . 18249311

