# Supplementary figures and images for: Vitamin D status contributes to the antimicrobial activity of macrophages against Mycobacterium leprae
Source: PLoS Negl Trop Dis. 2018 Jul 2;12(7):e0006608. doi: 10.1371/journal.pntd.0006608 (PMC6044553; doi:10.1371/journal.pntd.0006608)

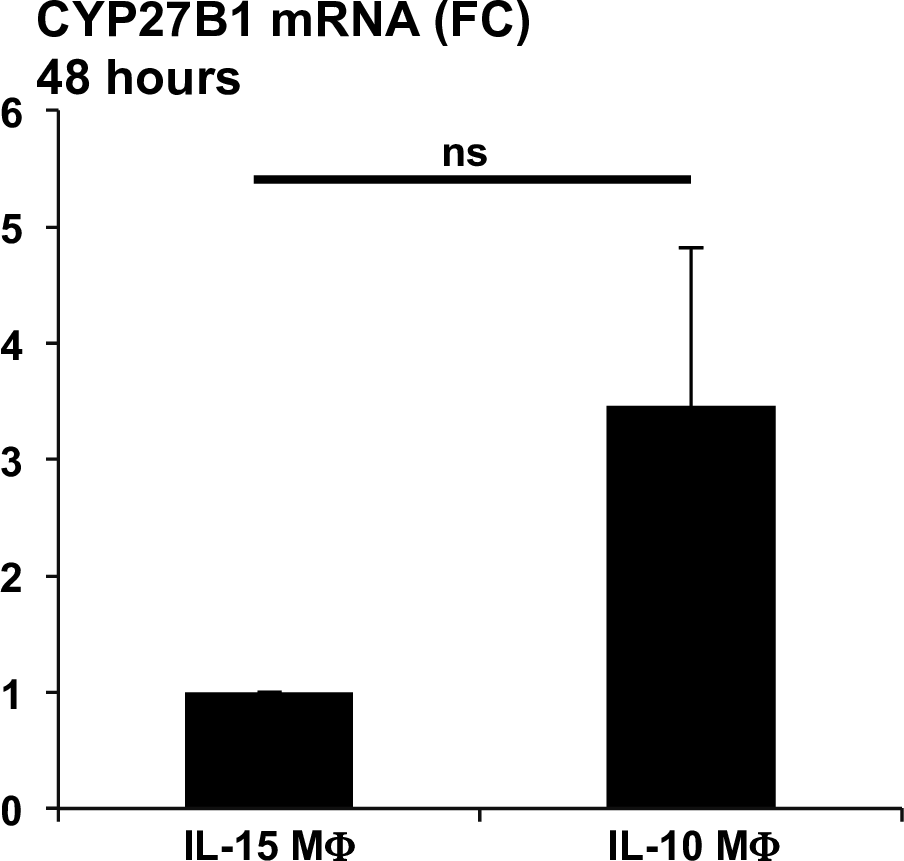

Supplement: S1 Fig — Primary human monocytes were treated with either IL-15 or IL-10 for 48 hours in SFM. No significant difference is observed between CYP27B1 mRNA expression levels between IL-15 MΦ and IL-10 MΦ. (TIF) [file pntd.0006608.s001.tif]
